# The influence of excluding patients with bystander return of spontaneous circulation in the current OHCA database

**DOI:** 10.1186/s12245-018-0197-4

**Published:** 2018-09-10

**Authors:** Hiroshi Otani, Ryo Sagisaka, Hideharu Tanaka, Hiroshi Takyu, Takahiro Hara, Toru Shirakawa, Shota Tanaka, Akira Maki

**Affiliations:** 1 Graduate School of Emergency Medical System, Kokushikan University, 7-3-1, Nagayama, Tama City, Tokyo 206-8515 Japan; 2 Japan Emergency Medical System Co. Ltd, Misato-Town, Miyazaki, Japan; 3 Research Institute of Disaster Management and EMS, Kokushikan University, 7-3-1, Nagayama, Tama City, Tokyo 206-8515 Japan

**Keywords:** First monitored rhythm, Bystander intervention, Public-access defibrillation, Cardiopulmonary resuscitation, Out-of-hospital cardiac arrest

## Abstract

**Background:**

The effect of bystander interventions has been extensively evaluated by cerebral function after 1 month post-resuscitation. However, patients who received bystander cardiopulmonary resuscitation (BCPR) and achieved the return of spontaneous circulation (ROSC) before the arrival of the emergency medical system (EMS) are routinely defined with an unknown electrocardiogram (ECG) and are usually excluded before analysis. The aim is to determine the influence of excluding patients with unknown first monitored rhythm, which includes cases of bystander ROSC, from the out-of-hospital cardiac arrest (OHCA) database.

**Methods:**

This nationwide population-based observational study was conducted in Japan using Utstein data from 2011 to 2014. In total, 91,995 patients with bystander-witnessed cardiogenic OHCA received resuscitation attempts in the pre-hospital setting. These patients were divided into three groups by the first monitored rhythm upon EMS arrival. We analysed the differences of datasets that included and excluded the unknown group and determined the effect on outcomes by multivariate logistic regression and odds ratios (ORs) with 95% confidence intervals (95% CIs).

**Results:**

When the unknown group was excluded from the data, the adjusted odds ratio (AOR) of cardiopulmonary resuscitation (CPR) to favourable cerebral performance category (CPC) 1 or 2 was decreased (conventional CPR: AOR, 1.90 to 1.58; chest-compression-only CPR: AOR, 2.08 to 1.69) compared to the unknown group’s inclusion. Conversely, the AOR of public-access defibrillation (PAD) was increased (AOR, 4.51 to 6.13).

**Conclusions:**

The exclusion of unknown ECGs from a dataset may lose ROSC patients by bystander CPR, causing selection bias to affect outcomes.

## Background

The increasing number of out-of-hospital cardiac arrest (OHCA) patients has become a global public health concern [1–4]. Several previous studies have shown that the two most important factors for patients with favourable neurological outcomes after OHCA were bystander cardiopulmonary resuscitation (BCPR) and public-access defibrillation (PAD) [2–6].

The effect of bystander interventions has been evaluated by cerebral function, such as cerebral performance category (CPC). However, the results of this evaluation depend on first monitored rhythms [7] categorised as follows: a shockable group that includes ventricular fibrillation (VF) and pulseless ventricular tachycardia (pVT); a non-shockable group that includes pulseless electrical activity (PEA) and asystole; and an unknown group that includes other conditions. The unknown group includes patients who received BCPR and achieved the return of spontaneous circulation (ROSC) before the arrival of emergency medical system (EMS) and are usually excluded before analysis.

Although Perkins et al. reported on new concepts of the first monitored rhythm and status of bystander interventions in greater detail in 2015. These have not been reflected internationally thus far [8]. Because a method for the handling of the unknown group has yet to be established, recent studies have included unknowns, excluded them or did not specify how they were managed [3–7, 9]. Furthermore, the potential bias of the unknown group has not been discussed. Therefore, we hypothesised that the exclusion of patients with unknown waveforms at EMS arrival resulted in a smaller effect of BCPR and PAD on favourable cerebral function than the inclusion of this group. This smaller effect would underestimate the effect of bystander resuscitation on patient outcomes.

The purpose of this study was to evaluate influence of excluding patients with bystander ROSC in the current OHCA Database according to Utstein-style guideline using multivariate logistic regression and estimating odds ratios (ORs) with 95% confidence intervals (CIs).

## Methods

### Study design

This population-based observational study was conducted using nationwide OHCA data collected in Japan from 2011 to 2014. The Utstein database was provided by Japan’s Fire and Disaster Management Agency (FDMA). The Institutional Review Board at Kokushikan University approved this study.

### Study setting

Japan encompasses approximately 378,000 km^2^ of land, and populated areas comprise 121,000 km^2^. According to the Statistics Bureau of the Ministry of Internal Affairs and Communications, the population of Japan in 2014 was approximately 127 million.

### EMS systems

The primary EMS system in Japan is provided by fire departments (FDs), and there were 752 FDs in 2014. All FDs are overseen by the FDMA. Almost every FD ambulance includes at least one emergency life-saving technician (ELST) who is qualified to provide advanced airway management, endotracheal intubation, intravenous lines, adrenaline administration, and defibrillation with a semi-automated defibrillator. EMS responders follow protocols provided by medical control councils for each region. These protocols are based on guidelines issued by the Japan Resuscitation Council.

### Study population

**Fig. 1:**
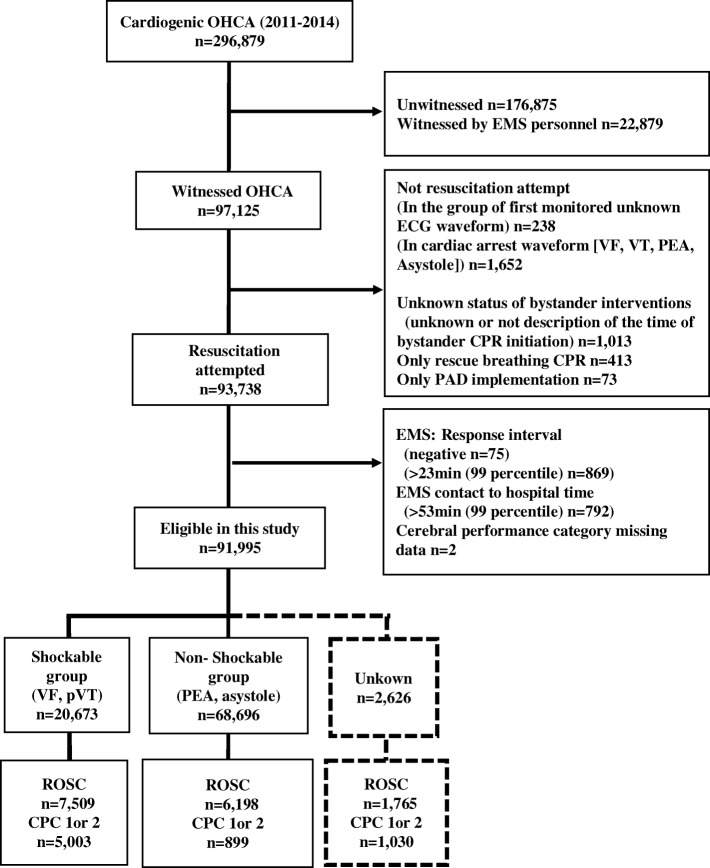
Study enrollment. OHCA data divided into three groups by initial ECG waveform at EMS arrival. Abbreviations: OHCA, out of hospital cardiac arrest; ECG, electrocardiogram; EMS, emergency medical service; ROSC, return of spontaneous circulation; PAD, public access defibrillation; response interval, call 119 to EMS contact with patient; unknown, first rhythm other than VF; pVT, PEA, asystole. VF, ventricular fibrillation; pVT, pulseless ventricular tachycardia; PEA, pulseless electrical activity. CPC, cerebral performance category

We sampled patients with presumed cardiogenic OHCA data registered from 2011 to 2014 according to Utstein-style guidelines in Japan. The exclusion criteria for this study were as follows: unwitnessed by laypeople, witnessed by EMS or fire department personnel, no CPR attempted. (1) Patients who are not ROSC on-scene without performing EMS resuscitation regardless of performing bystander CPR or not in the unknown ECG group. (2) Patients who are ROSC on-scene without received resuscitation attempt by both bystander and EMS in the unknown ECG group. (3) Although cardiac arrest waveform [VF, VT, PEA, asystole] is indicated at EMS arrival, resuscitation attempt is not performed by EMS on-scene, unknown status of bystander CPR or no description of the time of bystander CPR initiation, only rescue breathing CPR, only PAD implementation, response interval negative value, response interval > 23 min (99 percentile) and EMS contact to hospital arrival time > 53 min (99 percentile). A detailed inclusion/exclusion criterion is shown in Fig. 1.

All eligible patients were divided into three groups based on first monitored rhythm. The shockable group included patients with VF or pulseless VT. The non-shockable group included patients with PEA/asystole. Patients that were not applicable for either group were allocated into the unknown group.

### Data collection and quality control

In Japan, all OHCA data are prospectively collected by ELSTs or EMS personnel. All records are managed by the FDMA, and the results are published each year. The data are available as a part of the national OHCA registry; however, one must apply to the FDMA in advance to obtain the information. Kokushikan University applied and was granted these data. The records included the following data according to Utstein-style guidelines: prefectures, years, sex, age, bystander-witnessed status, types of bystander(s), bystander CPR, rescue breathing, use of an automated external defibrillator, first monitored rhythm, dispatcher assist, time course of resuscitation (e.g. time of patient collapse, receipt of Japanese emergency call for dispatch a fire engine or an ambulance (call 119), EMS contact with patients, EMS arrival on the scene, and hospital arrival), cause of cardiac arrest, return of spontaneous circulation in the pre-hospital setting, 1-month survival and neurological outcome 1 month after the event. The CPC was diagnosed by physicians. After 1 month, the outcome was followed up and described in all records by EMS personnel.

The act of performing chest compressions with rescue breathing by laypeople was called conventional CPR, and the use of only chest compressions was called chest-compression-only CPR. The response interval was defined as the time from the call to emergency services (119) to patient contact by EMS personnel. When laypeople delivered shocks using AED, the patient and their first recorded rhythms were regarded as the shockable group.

The Glasgow-Pittsburgh CPC scoring was defined as follows: category 1, good cerebral performance; category 2, moderate cerebral disability; category 3, severe cerebral disability; category 4, a coma or vegetative state; and category 5, death. Survival at 1 month post-resuscitation with a favourable neurologic outcome was defined as a CPC score of 1 or 2 [8, 10].

### Study endpoints

The primary outcome of our study was CPC 1–2 at 1 month post-resuscitation. Field ROSC was considered the secondary outcome of this study.

### Statistical analysis

#### Group characteristics

To compare the background factors, we divided the patients into three groups based on the first monitored rhythm. The proportions of qualitative variables were described as the number (%). Non-parametric continuous variables were calculated with the medians and interquartile ranges (IQR).

#### The differences of datasets that included and excluded the unknown group

We created two datasets that included and excluded the unknown group to assess differences in the effects of BCPR and PAD. To analyse the data objectively, we used multivariable logistic regression and adjusted for age, sex, types of bystander(s) (family, or others), types of CPR (none, conventional CPR, and chest-compression-only CPR), PAD, dispatcher assistance and response interval. Before calculating the adjusted ORs (AOR) with 95% CIs, crude ORs (COR) with 95% CIs were also estimated. In the logistic regression analysis, we confirmed that the continuous variable maintained the linearity for the outcomes.

All statistical analyses were performed using EZR version 2.3–1 (Saitama Medical Centre, Jichi Medical University, Saitama, Japan), a graphical user interface for R (R Foundation for Statistical Computing, Vienna, Austria) [11].

## Results

### Study participants

Figure 1 shows the patient selection process of the study. From 2011 to 2014, we sampled 296,879 patients with presumed cardiogenic OHCA in the database. A total of 91,995 patients were eligible for this study. The patients were divided into three groups based on first monitored rhythm: the shockable group (*n* = 20,673), the non-shockable group (*n* = 68,696), and the unknown group (*n* = 2626).

### Group characteristics

Table 1 shows the patient characteristics by group, as classified using first monitored rhythm. The median age of study patients was 79 years (IQR, 67–86), and the AED usage rate was 3.5% (3210 patients). Chest-compression-only CPR was the most common bystander intervention. More patients in the unknown group had favourable outcomes (39.2%, 1030 patients) than those in the shockable group (24.2%, 5003) and non-shockable group (1.3%, 899).

**Table 1:** Patient characteristics by study group classified using first monitored rhythm

Characteristics	All (*n* = 91,995)		Unknown rhythm (*n* = 2626)		Shockable group (*n* = 20,673)		Non-shockable group (*n* = 68,696)	
Year, no. (%)								
2011	22,339	(24.3)	665	(25.3)	5077	(24.6)	16,597	(24.2)
2012	22,566	(24.5)	575	(21.9)	5121	(24.8)	16,870	(24.6)
2013	23,285	(25.3)	696	(26.5)	5252	(25.4)	17,337	(25.2)
2014	23,805	(25.9)	690	(26.3)	5223	(25.3)	17,892	(26.0)
Age, median (25%, 75%)								
	79	(67, 86)	78	(66, 86)	67	(56, 77)	81	(72, 88)
Age category, no. (%)								
0–15 years	513	(0.6)	68	(2.6)	126	(0.6)	319	(0.5)
16–45 years	4338	(4.7)	152	(5.8)	2328	(11.3)	1858	(2.7)
45–74 years	31,562	(34.3)	820	(31.2)	11,770	(56.9)	18,972	(27.6)
≧ 75 years	55,582	(60.4)	1586	(60.4)	6449	(31.2)	47,547	(69.2)
Sex, no. (%)								
Male	55,993	(60.9)	1503	(57.2)	16,348	(79.1)	38,142	(55.5)
Types of bystanders, no. (%)								
Family member	57,600	(62.6)	1099	(41.9)	10,882	(52.6)	45,619	(66.4)
Bystander interventions, no. (%)								
Conventional CPR	9219	(10.0)	449	(17.1)	2766	(13.4)	6004	(8.7)
Chest-compression-only CPR	37,756	(41.0)	1482	(56.4)	9698	(46.9)	26,576	(38.7)
PAD	3210	(3.5)	0	(0.0)	3210	(15.5)	0	(0.0)
Dispatcher’s intervention, no. (%)								
Dispatcher assistance	43,671	(47.5)	1087	(41.4)	10,148	(49.1)	32,436	(47.2)
Response interval, median (25%, 75%)								
	8	(7, 10)	8	(6, 10)	8	(6, 10)	9	(7, 11)
Outcomes, no. (%)								
Field ROSC	15,472	(16.8)	1765	(67.2)	7509	(36.3)	6198	(9.0)
Favourable CPC	6932	(7.5)	1030	(39.2)	5003	(24.2)	899	(1.3)

Abbreviations: *CPR*, cardiopulmonary resuscitation; *PAD*, public-access defibrillation; *ROSC*, return of spontaneous circulation; *CPC*, cerebral performance category

*Conventional CPR*, CPR with rescue breathing; *chest-compression-only CPR*, CPR without rescue breathing

Response interval: the time from 119 call to EMS contact with patients

### The difference between datasets that included and excluded the unknown group

Table 2 compares the outcomes of a dataset that included the unknown group with one that excluded this group. The outcomes of patients with non-attempted BCPR are not included in this table. When the unknown group was excluded from the dataset, the proportion of patients with conventional CPR to CPC 1–2 is almost the same (inclusion 16.3%, 1127 patients; exclusion 16.2%, 958 patients). However, exclusion of this group resulted in 2.5% less patients with chest-compression-only CPR (inclusion 54.0%, 3742 patients; exclusion 51.5%, 3038 patients) and 3.2% more patients with PAD to CPC (inclusion 18.6%, 1286 patients; exclusion 21.8%, 1286 patients).

**Table 2:** The differences in outcomes between datasets that included and excluded the unknown group

	Field ROSC		CPC 1–2	
Dataset including unknowns	*n* = 15,472		*n* = 6932	
Conventional CPR	2135	13.8%	1127	16.3%
Chest-compression-only CPR	7237	46.8%	3742	54.0%
Overall PAD	1734	11.2%	1286	18.6%
Dataset excluding unknowns	*n* = 13,707		*n* = 5902	
Conventional CPR	1781	13.0%	958	16.2%
Chest-compression-only CPR	6112	44.6%	3038	51.5%
Overall PAD	1734	12.7%	1286	21.8%

Abbreviations: *PAD*, public access defibrillation; *ROSC*, return of spontaneous circulation; *CPC*, cerebral performance category; *conventional CPR*, CPR with rescue breathing; *chest-compression-only CPR*, CPR without rescue breathing

Not shown: patients with non-resuscitation attempts by bystanders

Table 3 shows the presumed effects of bystander interventions on ROSC and CPC 1–2 using multivariable analysis. In the dataset that included the unknown group, the AORs for CPC 1–2 were 1.90 for conventional CPR (95% CI, 1.73–2.08), 2.08 for chest-compression-only CPR (95% CI, 1.95–2.22) and 4.51 for PAD (95% CI, 4.12–4.94). In the dataset that excluded the unknown group, the AORs for CPC 1–2 were 1.58 for conventional CPR (95% CI, 1.43–1.75), 1.69 for chest-compression-only CPR (95% CI, 1.58–1.82) and 6.13 for PAD (95% CI, 5.57–6.74).

**Table 3:** The differences between datasets that included and excluded the unknown group

	Field ROSC				CPC 1–2			
	COR (95% CI)		AOR (95% CI)		COR (95% CI)		AOR (95% CI)	
Dataset including unknowns								
Conventional CPR	1.92	(1.82–2.03)	1.49	(1.40–1.58)	2.90	(2.69–3.13)	1.90	(1.73–2.08)
Chest-compression- only CPR	1.51	(1.46–1.57)	1.42	(1.36–1.48)	2.29	(2.17–2.42)	2.08	(1.95–2.22)
PAD	6.42	(5.97–6.90)	4.06	(3.75–4.39)	9.84	(9.13–10.6)	4.51	(4.12–4.94)
Dataset excluding unknowns								
Conventional CPR	1.69	(1.59–1.79)	1.21	(1.13–1.30)	2.73	(2.52–2.96)	1.58	(1.43–1.75)
Chest-compression- only CPR	1.34	(1.29–1.40)	1.19	(1.14–1.25)	2.03	(1.92–2.16)	1.69	(1.58–1.82)
PAD	7.28	(6.77–7.82)	5.24	(4.83–5.68)	11.8	(10.9–12.7)	6.13	(5.57–6.74)

Adjusted for the following confounding variables: age, sex, types of bystander(s) (others [reference], family), types of CPR (none [reference], conventional CPR, and chest-compression only CPR), PAD, dispatcher assistance, and response interval

In the group that included the unknown group, the AORs for ROSC were 1.49 for conventional CPR (95% CI, 1.40–1.58), 1.42 for chest-compression-only CPR (95% CI, 1.36–1.48) and 4.06 for PAD (95% CI, 3.75–4.39). In the group that excluded the unknown group, the AORs for ROSC were 1.21 for conventional CPR (95% CI, 1.13–1.30), 1.19 for chest-compression-only CPR (95% CI, 1.14–1.25) and 5.24 for PAD (95% CI, 4.83–5.68).

## Discussion

Using the Utstein database, this nationwide population-based observational study found that unknown first monitored rhythms are biased to outcomes. When the unknown group was excluded from the dataset, the effect of CPR(s) on favourable CPC decreased because of missing bystander ROSC. By excluding unknown rhythms, the effect of resuscitation by bystanders was underestimated. In addition, when this group was excluded, the effect of PAD on favourable CPC increased.

Since the American Heart Association (AHA) [12] released Guidelines 2000 for Cardiopulmonary Resuscitation, the dissemination of BCPR and PAD has rapidly increased internationally, and many studies related to bystander interventions have been published. In Japan, the effect of PAD has been evaluated since the use of AED by laypeople was permitted in 2004. Nakahara et al. reported that bystander interventions increased patients with favourable neurological outcomes and demonstrated that the implementation of BCPR and the use of AED increased [2]. In addition, Kitamura et al. reported that AED use for VF patients by laypeople was associated with an increased number of survivors with favourable neurological outcomes in Japan [3, 4].

In these studies, and several others, the method of handling patients with unknown first monitored rhythm was separated into two patterns. Several studies excluded or obscured how these unknown data were managed because of undocumented details [3–5, 7, 13–15]. Other studies included this data [2, 6, 9].

Cummins et al. posited that waveforms other than VF, pulseless VT, PEA, and asystole included the following characteristics: (1) some electrical activity was observed in a cardiac arrest patient; (2) ventricular escape complexes represented the last electrical activity; and (3) electro-mechanical dissociation, which is currently considered PEA [10, 16]. However, unknown ECG was not completely defined.

In 2015, Perkins et al. reported on new concepts of first monitored rhythms, which included VF/pVT, PEA/asystole, bradycardia, AED non-shockable, AED shockable and not-recorded/unknown [8]. Globally, these categories are not presently in widespread use. Therefore, the unknown group still has extensive variation, which makes international comparisons difficult. Even in recent studies, the first monitored unknown rhythm remains ambiguous.

In this study, we found 6932 patients who had favourable outcomes, of which 14.9% had unknown first monitored rhythm in the dataset that included the unknown group. Furthermore, we confirmed that 11.4% of the unknown group achieved ROSC in the pre-hospital setting. Thus, the unknown group had a relatively greater number of patients with favourable outcomes. In addition, when we excluded the unknown group, the effect of CPR (both conventional and chest-compression-only) to favourable outcome decreased. Because the CPR implementation rate in the unknown group was the highest of the three groups and many of these patients had favourable outcomes, this result suggests that excluding the unknown group decreases the total number of CPR cases. As a result, the odds ratio of CPR for favourable CPC decreased (conventional CPR: AOR, 1.90 to 1.58; chest-compression-only CPR: AOR, 2.08 to 1.69). This result indicates that studies that exclude the unknown group miss patients who received BCPR and achieved ROSC before EMS arrival, leading to underestimated effects of resuscitation by bystander.

In the dataset that included the unknown group, 18.6% of the patients with attempted BCPR had favourable outcomes by PAD shocks. However, in the dataset that excluded the unknown group, 21.8% of patients had favourable outcomes by PAD shocks. Although this change was seemingly benign at first glance, the odds ratio of PAD for favourable CPC significantly increased after excluding the unknown group (AOR, 4.51 to 6.13). This result also suggests that the unknown group had a selection bias to the outcome.

We also carried out the subgroup study of differences of datasets that included and excluded the unknown group in order to assess the influence of the group who received BCPR without PAD and no EMS resuscitation (Table 4). We excluded these 1037 patients from unknown group. The ORs of CPR and PAD in this group became close to the ORs of excluding data set (subgroup study of conventional CPR: AOR, 1.53 to 1.58; chest-compression-only CPR: AOR, 1.67 to 1.69, PAD: 5.80 to 6.13). Therefore, we concluded that these patients had bias to the outcomes. However, since these patients may have been in cardiac arrest or no cardiac arrest and it has great influence on the outcomes, how to assess these bystander efforts should be sought and discussed. In addition, this result also addressed that other 1589 patients of unknown group could be available for OHCA analysis because they are cardiac arrest.

**Table 4:** Subgroup of the differences between datasets that included and excluded the unknown group

	Field ROSC				CPC 1–2			
	COR (95% CI)		AOR (95% CI)		COR (95% CI)		AOR (95% CI)	
Dataset including unknowns (excluding patients who received BCPR without PAD and no EMS resuscitation)								
Conventional CPR	1.68	(1.59–1.78)	1.24	(1.16–1.32)	2.57	(2.38–2.79)	1.53	(1.39–1.69)
Chest-compression-only CPR	1.35	(1.30–1.40)	1.22	(1.16–1.27)	1.98	(1.87–2.09)	1.67	(1.56–1.79)
PAD	6.94	(6.46–7.46)	4.91	(4.53–5.32)	11.1	(10.3–12.0)	5.80	(5.28–6.37)
Dataset excluding unknowns								
Conventional CPR	1.69	(1.59–1.79)	1.21	(1.13–1.30)	2.73	(2.52–2.96)	1.58	(1.43–1.75)
Chest-compression-only CPR	1.34	(1.29–1.40)	1.19	(1.14–1.25)	2.03	(1.92–2.16)	1.69	(1.58–1.82)
PAD	7.28	(6.77–7.82)	5.24	(4.83–5.68)	11.8	(10.9–12.7)	6.13	(5.57–6.74)

Adjusted for the following confounding variables: age, sex, types of bystander(s) (others [reference], family), types of CPR (none [reference], conventional CPR, and chest-compression only CPR), PAD, dispatcher assistance, and response interval

To accurately assess the efforts of bystander resuscitation and decrease potential bias, it is essential that future studies evaluate the quality of BCPR, verify if patients are cardiac arrest and adhere to the new guidelines. The interpretation of the unknown group may be different depending on country or region. Therefore, we recommend that studies evaluating the bystander effect clearly report the method of handling the unknown group to provide the transparency until official guidelines of each countries define the unknown first monitored rhythm. We suggest four exclusion criteria of unknown ECGs from OHCA data: (1) no ROSC without performing EMS resuscitation, or (2) ROSC without any resuscitation attempt by both bystander and EMS, or (3) rhythm available and no EMS resuscitation, or (4) ROSC by BCPR without PAD and no EMS resuscitation. These criteria must be used until the new guidelines are applied to each country.

### Study limitations

Because of the observational study, unknown bias could have affected its results, such as patient backgrounds and in-hospital care, both of which are not included in the Japanese Utstein database. These factors may have influenced the survival outcomes. Furthermore, we were unable to obtain the actual unknown ECG data, as they were not described in the Japanese Utstein database. Therefore, it requires a further nationwide investigation for acquiring them, and we consider that it will be the next examination of this study. Defibrillation time from collapse was not considered due to the lack of data. In addition, the location of cardiac arrest was available [17–19], but we could not include the location where PAD was delivered. Moreover, we included the BCPR group, which may have affected outcomes because the quality of CPR was not considered. Assessing the quality of BCPR prior to EMS personnel arrival was not realisable under the present system. Finally, our results cannot be generalised to children nor non-cardiogenic or unwitnessed OHCA patients.

## Conclusions

Studies that exclude unknown ECGs may lose patients who received BCPR that achieved field ROSC and favourable CPC, leading to selection bias in outcomes. To minimise the underestimation of the bystander effect and potential bias of unknown ECGs, future studies analysing OHCA should carefully manage and report the method of handling these missing data.
